# Additive and non-additive genetic variance in juvenile Sitka spruce (*Picea sitchensis* Bong. Carr)

**DOI:** 10.1007/s11295-023-01627-5

**Published:** 2023-11-08

**Authors:** J.J. Ilska, D.J. Tolhurst, H. Tumas, J. P. Maclean, J. Cottrell, S.J. Lee, J. Mackay, J.A. Woolliams

**Affiliations:** 1 The Roslin Institute, Royal (Dick) School of Veterinary Science, University of Edinburgh, Easter Bush, Midlothian, Scotland EH25 9RG UK; 2 Department of Biology, University of Oxford, South Parks Road, Oxford, OX1 3RB UK; 3 Forest Research, Northern Research Station, Roslin, Midlothian, EH25 9SY UK; 4 Present Address: The Kennel Club, 10 Clarges St, London, W1J 8AB UK; 5 Present Address: Department of Forest and Conservation Sciences, University of British Columbia, 2424 Main Mall, Vancouver, BC V6T 1Z4 Canada; 6 Present Address: Norwegian University of Life Sciences, Postboks 5003, 1432 Ås, Norway

**Keywords:** Epistasis, Genetic variance, Non-additive variation, RADseq, Single-step, Sitka spruce, Spatial model

## Abstract

Many quantitative genetic models assume that all genetic variation is additive because of a lack of data with sufficient structure and quality to determine the relative contribution of additive and non-additive variation. Here the fractions of additive (*f*_*a*_) and non-additive (*f*_*d*_) genetic variation were estimated in Sitka spruce for height, bud burst and pilodyn penetration depth. Approximately 1500 offspring were produced in each of three sib families and clonally replicated across three geographically diverse sites. Genotypes from 1525 offspring from all three families were obtained by RADseq, followed by imputation using 1630 loci segregating in all families and mapped using the newly developed linkage map of Sitka spruce. The analyses employed a new approach for estimating *f*_*a*_ and *f*_*d*_, which combined all available genotypic and phenotypic data with spatial modelling for each trait and site. The consensus estimate for *f*_*a*_ increased with age for height from 0.58 at 2 years to 0.75 at 11 years, with only small overlap in 95% support intervals (*I*_95_). The estimated *f*_*a*_ for bud burst was 0.83 (*I*_95_=[0.78, 0.90]) and 0.84 (*I*_95_=[0.77, 0.92]) for pilodyn depth. Overall, there was no evidence of family heterogeneity for height or bud burst, or site heterogeneity for pilodyn depth, and no evidence of inbreeding depression associated with genomic homozygosity, expected if dominance variance was the major component of non-additive variance. The results offer no support for the development of sublines for crossing within the species. The models give new opportunities to assess more accurately the scale of non-additive variation.

**Supplementary Information:**

The online version contains supplementary material available at 10.1007/s11295-023-01627-5.

## Introduction

Breeding is well established in many forest tree species but it is often hindered by the lengths of generation intervals. Current breeding cycles in conifers, including spruces, pines, larches and firs, commonly exceed 15 years requiring candidate trees to have reached reproductive age and have sufficiently accurate predictions of breeding value obtained from early predictors of adult performance, possibly supplemented with progeny testing, prior to selection decisions being made. The history of Sitka spruce (*Picea sitchensis* [Bong.] Carr.) in the UK is an example of a well-planned and executed breeding programme which is faced with this challenge of long intervals. Sitka is a conifer species originating from the Pacific North West extending from south-eastern Alaska to northern California. It was first brought to the UK in the 1830s (Lee et al. 2013), and now accounts for over 50% of all the area planted with conifers and ~25% of all woodland area of Great Britain (IFOS-Statistics, 2022). Breeding objectives for the species relate to its primary use for construction timber and wood pulp (Lee et al. 2013), and although improvements have been achieved since the start of plus-tree selection in the early 1960s, only two cycles of selection have been completed (Lee and Connelly, 2010). This time constraint along with the high cost of field evaluations, among others, has often limited the ability to characterise fully the genetic control of phenotypic traits, such as their partitioning into additive and non-additive gene effects.

An attraction of genomic prediction is the potential to transform forest breeding through reducing generation intervals or increasing selection intensity while retaining sufficient accuracy of the predicted breeding values (EBVs) to obtain faster rates of improvement (Grattapaglia, 2017). This is due to a different approach to estimating breeding values using molecular data (Meuwissen et al. 2001) and genomic relationship coefficients (Van Raden, 2008), compared to using pedigree and the matrix of numerator relationship coefficients derived from it. In the pedigree approach, the breeding values are predicted from models that, beyond the base generation, rely on estimating Mendelian sampling terms of individuals, which in turn relies on obtaining phenotypic information on the candidate or offspring. In contrast, when applying the molecular approach, the breeding values are predicted from the estimated effects of (dense and genome wide) marker alleles, typically SNPs, which can be obtained for all genotyped individuals provided relevant data are available for estimating the SNP effects. With genotypes available from ‘conception’, one barrier to reducing the generation interval and obtaining an EBV that encompasses an individual’s own genome is removed.

Most attention in tree breeding (as in other fields) has focused upon developing genomic predictions of breeding value, i.e. additive genetic merit, and it is the variance of the breeding values that defines the additive genetic variance. However, the total genetic variance includes contributions from non-additive genetic variation, and predicting non-additive effects can be used to improve the merit of those deployed in the wider forest population for timber. The molecular data make it possible to access the non-additive genetic effects more directly and to predict non-additive components of the total genetic merit (Vitezica et al. 2017; Joshi et al. 2020). One benefit of using the genomic data is that it is feasible to estimate non-additive genetic variance from simpler designs than would be necessary using pedigree data. In forestry, the ease of vegetative propagation allows clonal experiments to be established which provide material to estimate the total genetic variance and broad heritability (*H*^2^), while the genotypic data can be used to estimate genomic relationships, and hence estimate the additive genetic variance and narrow sense heritability (*h*^2^). Consequently, the extent and potential impact of the non-additive genetic variance can be estimated.

One of the challenges of advancing the use of genomic techniques in Sitka has been the need to generate the thousands of SNP marker genotypes on selection candidates to provide a marker coverage of the genome that is sufficiently dense. There are multiple ways of obtaining SNP genotypes, e.g. through SNP chip arrays, whole genome resequencing, or reduced representation sequencing. Restriction-site-associated DNA sequencing (RADseq) belongs to a group of reduced representation sequencing methods that have been particularly popular in non-model species. The benefits of RADseq are its flexibility and relatively low cost compared to whole genome resequencing (Parchman et al. 2018) but it is particularly attractive for species, including many conifers, with large repetitive genomes where the compilation of a draft reference genome is challenging (Pan et al. 2015; Fuentes-Utrilla et al. 2017; Parchman et al. 2018). While assays for RADseq have been described for Sitka (Fuentes-Utrilla et al. 2017), this was for a single family and their application and performance across multiple families are unknown. One of the drawbacks of RADseq is the stochastic nature of the sequence reads for a given coverage, particularly when the coverage is low but this can be overcome using imputation (e.g. Li et al. 2009).

The primary goal of this paper was to estimate the fractions of additive and non-additive genetic variance in the total genetic variance of Sitka spruce, based on SNP markers derived from RADseq data and phenotypic data collected on height, pilodyn depth and bud burst in the offspring of three full sib families. Height and pilodyn depth are important traits for the tree breeder as they are key in determining quantity and quality of timber, while bud burst is an adaptive trait that provides insight into local adaptation to climatic conditions. The newly developed linkage map of the Sitka spruce genome (Tumas et al. 2023) allowed the application of an imputation procedure, which enabled missing genotypes to be recovered, thereby making maximum use of the available SNP data. The tree height data was collected at different ages and allowed the sensitivity to site and family variation to be studied as it came from three large sib families, clonally replicated across three geographically and climatically diverse sites. The analyses employed spatial modelling to account for natural and extraneous variation within each site (Gilmour et al. 1997). To the authors’ knowledge, this is the first paper where the heritability of economically important traits in conifers was estimated using analyses that simultaneously accounted for additive and non-additive genetic variance based on genomic data along with spatial modelling.

## Materials and methods

### Population

The phenotypic and genomic data were based on material in a large field experiment established in 2005 by Forest Research (https://www.forestresearch.gov.uk). The experiment was designed as three full-sib families (denoted as SF1, SF2 and SF3) each with 1500 offspring (where offspring represents a unique genotype), clonally replicated across three contrasting sites: Huntly, Llandovery and Torridge (Table 1). The families were created by controlled pollination of maternal clones growing in the Sitka spruce clone bank of Forest Research. The genomic data presented below revealed that SF3 was not a single full-sib family but was a mix of two full-sib families with a common maternal parent, in an approximate ratio of 3:2. The parents of both SF1 and SF2 and 2 of the 3 parents of SF3 were unrelated members of the Forest Research Sitka Spruce Breeding Population but were otherwise unselected. The source of the unknown parent in SF3 is uncertain, but was not another of the genotyped parents.

**Table 1:** Geographic and climatic characteristics of the three sites. The accumulated temperature is defined by the number of days above 5°C using historical data from the UK Meteorological Office over the 30-year recording period 1961–1990

Characteristic	Units	Huntly	Llandovery	Torridge
		Scotland	Wales	England
Latitude	°N	57.58	51.97	50.82
Longitude	°W	2.82	4.12	4.37
Height above sea level	m	140	230	120
Accumulated temperature	°days	1106	1450	1828

Each offspring was vegetatively propagated from cuttings to produce 12 ramets (clonally replicated copies of an offspring genotype), with four ramets of each genotype on each site and, within sites, one ramet of each genotype in each of four replicate blocks. In addition to the intended 1500 offspring trees, each block contained 46 control trees raised from open pollinated seed collected from Haida Gwaii (formerly Queen Charlotte Islands), British Columbia. Control trees were not cloned. The trees of SF1 at Torridge formed the data for a previous publication (Fuentes-Utrilla et al. 2017).

### Traits

All trees had their height measured after 2, 4, 6 and 11 years of age, with the ages determined by available resources. In addition, the depth of penetration of a pilodyn pin at breast height after 10 years was recorded using a Pilodyn 6J Forest device (Proceq, Switzerland) as an indicator trait for wood density, but due to resource constraints only at the Torridge site. Trees from family SF1 were also scored for the timing of bud-burst at the start of the fifth year in 2009 following the scale of Krutzsch (1973) at all sites, where 0 is a dormant bud, 1 is slightly swollen, up to 8 where all needles are more or less spread with new buds developing. This scoring was carried out on three occasions at each site: 5A on 29/04, 06/05 and 06/05 at Torridge, Huntly and Llandovery; 5B on 06/05, 12/05 and 12/05; and 5C on 12/05, 19/05 and 20/05 respectively. A summary of the design for the measurement of traits is shown in Table 2. The number of trees available for measurement of height at each age is shown in Table 3, which also provides a guide to numbers of trees assessed for bud burst and pilodyn measurements at the intermediate ages. Table 4 shows the raw means and standard deviations for all traits measures at each site for each family including both genotyped and ungenotyped offspring.

**Table 2:**
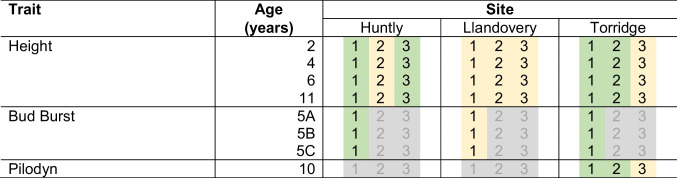
The design of trials showing which traits were measured at which sites and in which families (SF1, 2 or 3). Measurements were made for all trials other than those shaded grey. Trials shaded green are those which have one master block while trials shaded yellow have two master blocks (described in Supplementary Information 3)

**Table 3:** Numbers of trees measured for height according to site, age and family, together with the total number of offspring, and the number of genotyped offspring represented

Age (years)	Huntly			Llandovery			Torridge		
	SF1	SF2	SF3	SF1	SF2	SF3	SF1	SF2	SF3
*Trees*									
2	5854	5903	5670	5786	5805	5805	5988	5981	5845
4	5698	5861	5400	5220	5288	5164	5987	5875	4207
6	5676	5857	5400	4716	4900	4527	5982	5829	4089
11	5673	5856	5396	4326	4760	3947	5982	5829	4076
*Offspring*									
2	1500	1500	1496	1500	1500	1496	1500	1500	1496
4	1500	1500	1496	1498	1500	1496	1500	1500	1495
6	1500	1500	1496	1493	1500	1491	1500	1500	1495
11	1500	1500	1495	1488	1499	1467	1500	1500	1495
*Genotyped*									
2	573	474	478	573	474	478	573	474	478
4	573	474	478	572	474	478	573	474	478
6	573	474	478	569	474	477	573	474	478
11	573	474	478	569	474	470	573	474	478

**Table 4:** The raw means and standard deviations (S.D) for all traits measured at all sites within families SF1, SF2 and SF3 including both genotyped and ungenotyped offspring

Site	Trait	Units	Age	Family SF1		Family SF2		Family SF3	
				Mean	S.D.	Mean	S.D	Mean	S.D
Huntly	Height	*m*	2	0.708	0.108	0.616	0.117	0.662	0.107
			4	1.374	0.294	1.431	0.272	1.369	0.312
			6	2.831	0.584	2.855	0.458	2.760	0.550
			11	7.612	1.023	7.573	1.306	8.106	1.302
	Bud burst	*units*	5A	2.893	0.483	-	-	-	-
			5B	5.564	0.655	-	-	-	-
			5C	6.650	0.603	-	-	-	-
Llandovery	Height	*m*	2	0.673	0.118	0.658	0.160	0.647	0.109
			4	1.319	0.372	1.417	0.430	1.295	0.358
			6	2.674	0.777	2.640	0.753	2.256	0.601
			11	6.691	1.391	5.848	1.589	4.549	1.235
	Bud burst	*units*	5A	3.945	0.687	-	-	-	-
			5B	4.505	0.688	-	-	-	-
			5C	5.104	0.610	-	-	-	-
Torridge	Height	*m*	2	0.759	0.128	0.676	0.107	0.606	0.099
			4	1.613	0.351	1.364	0.311	1.024	0.344
			6	3.365	0.615	2.809	0.669	2.054	0.697
			11	8.634	0.813	7.815	1.177	6.292	1.303
	Bud burst	*units*	5A	2.548	0.738	-	-	-	-
			5B	3.355	1.055	-	-	-	-
			5C	5.150	1.082	-	-	-	-
	Pilodyn	*mm*	10	14.835	1.644	17.214	2.151	14.075	2.192

### RADseq genotypes

#### Assay

The RADseq data used for SF1 were initially produced for Noveltree (https://cordis.europa.eu/project/id/211868), and those for SF2 and SF3 were produced for Procogen (https://cordis.europa.eu/project/id/289841). DNA was extracted from the needles of the 6 known parents and from randomly selected subsets of the offspring in each family. The protocols for DNA extraction and RADseq digestion were fully described in Fuentes-Utrilla et al. (2017). Briefly, DNA was extracted using a Qiagen DNeasy Plant mini-kit but with the protocol modified to maximise DNA quantity. The extracted DNA was then subjected to a double-digest RADseq protocol using *AlnwI* and *PstI* enzymes. Paired-end reads were produced for parents, and single-end for offspring using Illumina HiSeq2000. While the protocol for the RADseq digestion was the same for all 3 families, the resulting average read length ranged from 45bp in offspring of SF1 to 112bp in parents of SF2 and SF3. In addition to the six known parents, the numbers assayed in each family were 578, 478 and 486 from SF1, SF2 and SF3 respectively.

#### Bioinformatic pre-processing of RADseq data

The raw, barcoded fastq-libraries were de-multiplexed using RADtools v1.2.4 (Baxter et al. 2011). The paired-end reads of parents were then screened for PCR duplicates using a Perl script (Kerth, 2014) which removed 22–24% reads in parents of SF1 and 43–46% reads in parents of SF2 and SF3. Offspring whose number of reads fell outside 3 standard deviations from the overall mean within each family were removed, and this resulted in the removal of 5, 4 and 8 offsprings from families SF1, SF2 and SF3 respectively. Adapter sequences were removed from all reads using Scythe v.0.994 (Buffalo, 2014). The reads were then processed with the ‘process radtags’ package of Stacks v.2 (Rochette and Catchen, 2017) to remove reads with uncalled bases and quality scores <20, and then to truncate all reads to 45bp to allow simultaneous processing of all three families. The ‘k-mer filter’ option of Stacks v.2 was used to remove both abundant and rare k-mers, with the default k-mer size set to 15. The final number of reads retained for further analysis ranged between 17.4 and 20.2M for each of the six genotyped parents, and between 2.2 and 3.5M reads for each of the 1525 remaining offspring.

#### SNP genotyping

SNP markers were identified and genotyped using the Stacks v.2 pipeline: ‘ustacks’ to build loci within a sample; ‘cstacks’ to construct a catalogue of loci from parental samples; ‘sstacks’ to match loci from all samples to the catalogue; ‘tsv2bam’ to transpose data to become locus oriented; ‘gstacks’ to call variants sites and genotyping individuals. The parameters used for the genotyping were optimised as recommended in Paris et al. (2017). The outcome was as follows: minimum stack depth (*m*) set to 2, distance between stacks (*M*) set to 3 and distance between catalogued loci (*n*) set to 3. The resulting genotypes were exported to a ‘vcf’ format using the ‘populations’ package of Stacks v.2, parameterised so that a locus was processed if it was detected in at least 3 populations (*p*=3), and in at least 3% of all individuals across all populations (*R*=3). The parameter settings were chosen to minimise the number of Mendelian inconsistencies and missing values across the resulting genotypes.

#### SNP quality control

The genotypes were split into 3 within-family datasets using Plink (Purcell et al. 2007). Quality control was then applied within each family by sequentially removing individuals with call rates less than 0.6 and then removing SNPs with call rates less than 0.8 and MAF<0.15. The choice of 0.15 was guided by noting that within full-sib families, the segregating SNPs are expected to have frequencies of either 0.25, 0.5 or 0.75 and, within the full-sib families that were anticipated, an observed MAF<0.15 would be highly unusual. The resulting call rates across all retained individuals and SNPs were 0.77, 0.79 and 0.81 for SF1, SF2 and SF3 respectively. Mendelian inconsistencies were examined using a custom Python script (https://github.com/joannailska/Mendelian_inconsistencies). Firstly, the observed genotype frequencies were compared to the expected frequencies conditional on the parental genotypes. SNPs were removed if the corresponding *χ*^2^-test exceeded the *P*<0.05 threshold having applied a Bonferroni correction for the number of SNPs. Secondly, the stochastic nature of sequence reads results in a background number of opposing homozygotes in offspring and parent, and offspring genotypes were removed if incompatible, given the manifold greater coverage of the parents. The summary of the latter step identified anomalies for 194 of the 478 offspring in SF3. This led to the discovery of the family structure of SF3 being a mix of two full-sib families, denoted SF3A and SF3B, with a common maternal parent, and SF3A comprising a larger fraction (0.594, s.e. 0.022) of the offspring (see Supplementary Information 1). The numbers of SNPs by family are reported in Table 5. Among the retained SNPs, 2054 SNPs were segregating in all three families and offered an element of validation, and these are henceforth referred to as ‘common SNPs’.

**Table 5:** The number of SNP markers and trees retained within each family (SF1, SF2 or SF3) following quality control, together with the percentage of these SNPs (i) among the 2054 SNPs found in all three families (‘common’) and (ii) among the 1630 SNPs that were both ‘common’ and mapped and used for imputation

	Family		
	SF1	SF2	SF3
SNPs	15,452	17,915	13,176
% present in all SF	13.2	11.5	15.6
% present in all SF and mapped	10.5	9.1	12.4
Offspring	573	474	478

#### Imputation

Among common SNPs, 1630 (78%) had been reliably assigned to the 12 linkage groups of the linkage map compiled by Tumas et al. (2023) and this map was used for imputation. For each of the three families used in this study, the available genotyping data for these 1630 loci were assigned to the 12 linkage groups and ordered within them. The genotypes identified were processed using AlphaPeel (Whalen et al. 2018, v.1.1.0 for SF1 and SF2, v.2.1.0 for SF3) using the multi-locus peeling option, with an additional parameter giving the map distance in Morgans spanning the loci for each linkage group. A dummy identity was assigned to the unknown paternal parent of SF3B. The distribution of SNPs across linkage groups is shown in Table 6. Imputation accuracy was assessed using posterior genotype probabilities provided by AlphaPeel for the full-sib offspring and summarised by assuming genotypes to be assigned if the posterior probability for a genotype was greater than *p* and varying *p* over the range 0.5 to 1 (so the assignment is unique). For a given value of *p*, the SNP call rate over offspring and offspring call rates over SNPs were calculated. An additional assessment described in Supplementary Information 2 estimated the probability of error in genotype assignment to be 0.013.

**Table 6:** The partition of the 1630 ‘common’ and mapped loci (# SNP) and their average spacing (#SNP/cM) across linkage groups

	Linkage group												Overall
	1	2	3	4	5	6	7	8	9	10	11	12	
Length (cM)	218	194	201	194	165	174	203	199	164	157	128	146	2143
# SNP	159	163	153	146	109	139	149	129	125	129	108	121	1630
# SNP/cM	0.73	0.84	0.76	0.75	0.66	0.80	0.73	0.65	0.76	0.82	0.84	0.83	0.76

### Statistical models

A site and family combination is hereafter referred to as a trial (with 9 trials in total). Each trial was designed as a randomised complete block design with four replicate blocks. Each block was nominally allocated 1500 offspring trees and the controls. Given the objectives, the controls were treated as filler plots with missing phenotypes in the analysis. Due to topographic constraints, some blocks were spatially separated (non-contiguous) which required ‘master blocks’ to be constructed. For trials with non-contiguous blocks, two master blocks were created and filler plots with missing phenotypes were added to ensure a continuous spatial structure within each master block. Trials with contiguous blocks were treated as having a single master block. The number of master blocks in each trial is shown in Table 2, with further information provided in Supplementary Information 3.

All models were fitted separately for each trial and included: (i) a preliminary spatial model without genomic data and (ii) an extended spatial model with genomic data. The novel feature of (ii) is that phenotypic data was included on all offspring trees (regardless of whether they have been genotyped) while genomic data was also included on the subset of genotyped trees in each family. For SF3, all data was included for both SF3A and SF3B offspring, genotyped or otherwise, as described in the following sections. This preserved all data to estimate the genetic and non-genetic variance parameters, and enabled estimates of additive and non-additive genetic variance parameters to be obtained. A similar approach was proposed by Tolhurst et al. (2019), but note that here the ungenotyped trees are utilised to estimate the total genetic variance, rather than just the non-genetic variances.

#### Preliminary spatial model

The preliminary spatial model fitted was a univariate BLUP model that accommodated the experimental design and spatial modelling for each trial. This linear mixed model for **y**, the vector of available phenotypic data on the 1500 offspring trees in each trial, can be written as:

1 $$\textbf{y}=\textbf{Xb}+{\textbf{Z}}_u\textbf{u}+{\textbf{Z}}_v\textbf{v}+\textbf{e}$$

where **b** is a vector of fixed effects, here only the mean, with **X** being a vector 1’s, **u** is a vector of random genetic effects for all offspring trees with design matrix **Z**_*u*_, **v** is a vector of random non-genetic effects, here only blocks, with design matrix **Z**_*v*_, and **e** is the vector of residuals. The genetic effects are assumed to be distributed as **u** ~ MVN(**0**, ${\upsigma}_u^2\textbf{I}$), where ${\upsigma}_u^2$ is the total genetic variance. The block effects are assumed to be distributed as **v** ~ MVN(**0**, ${\upsigma}_v^2\textbf{I}$), where ${\upsigma}_v^2$ is the block variance. The residuals are assumed to be distributed as **e** ~ MVN(**0**, **R**), where **R** is the residual variance matrix which includes a model for natural and extraneous variation, i.e. variation due to random error and spatial trend (Gilmour et al. 1997). The residual variance matrix is given by:

2 $$\textbf{R}={\upsigma}_r^2\textbf{I}+{\upsigma}_s^2{\oplus}_{k=1}^b{\boldsymbol{\Sigma}}_{c(k)}\left({\rho}_c\right)\otimes {\boldsymbol{\Sigma}}_{r(k)}\left({\rho}_r\right)$$

where ${\upsigma}_r^2$ is the random error variance and ${\upsigma}_s^2$ is the spatial error variance, such that ${f}_r={\sigma}_r^2/\left({\sigma}_r^2+{\sigma}_s^2\right)$ and ${f}_s={\sigma}_s^2/\left({\sigma}_r^2+{\sigma}_s^2\right)$ are the fractions of random and spatial error variance. The Kronecker plus operator (⊕) constructs a block-diagonal model across the *b* master-blocks (*b* = 1 or 2; Table 2) and the Kronecker product operator (***⊗***) constructs a separable model between the columns and rows in each master block. Note that the model for each master block is parameterised by different auto-correlation matrices, i.e. **Σ**_*c*(*k*)_ and **Σ**_*r*(*k*)_, but the same auto-correlation parameters, *ρ*_*c*_ and *ρ*_*r*_. This constructs a common spatial model for all master blocks within a trial. The significance of the spatial models was informally assessed by log-likelihood ratio tests and the Akaike Information Criterion, and showed considerable improvements compared to models with independent residuals, i.e. **e** ~ MVN(**0**, ${\upsigma}_e^2\textbf{I}$). The model described by Eqs. (1) and (2) is hereafter referred to as model 1.

#### Extension to include genomic data

Model 1 was then extended to genomic BLUP using a genomic relationship matrix, **G**, derived from RADseq data (described below). This model included phenotypic data on all offspring trees, while genomic data were included on the subsets of genotyped trees in SF1, SF2 and SF3 respectively. Let the vector of genetic effects be partitioned as ***u =*** (***u***_1_^T^, ***u***_2_^T^)^T^ where ***u***_1_ and ***u***_2_ are the vectors for the ungenotyped and genotyped trees, respectively. Since there was clonal replication, the genetic effects for the genotyped trees can be further partitioned into additive and non-additive effects, where ***u***_2_ = ***u***_2(*a*)_ + ***u***_2(*d*)_. The design matrix is partitioned conformably with ***u***, where **Z** = [**Z**_1_ **Z**_2_].

The linear mixed model for **y** can now be written as:

3 $$\textbf{y}=\textbf{Xb}+{\textbf{Z}}_1{\textbf{u}}_1+{\textbf{Z}}_2\left[{\textbf{u}}_{2(a)}+{\textbf{u}}_{2(d)}\right]+{\textbf{Z}}_v\textbf{v}+\textbf{e}$$

where the non-genetic and residual terms are as described for model 1. The genetic effects for the ungenotyped trees are assumed to be distributed as **u**_1_ ~ MVN(**0**, ${\upsigma}_u^2\textbf{I}$). The additive genetic effects (breeding values) for the genotyped trees are assumed to be distributed as **u**_2(*a*)_ ~ MVN(**0**, ${\upsigma}_a^2\textbf{G}$), where ${\upsigma}_a^2$ is the additive genetic variance and **G** is the genomic relationship matrix. The non-additive effects for the genotyped trees are assumed to be distributed as **u**_2(*d*)_ ~ MVN(**0**, ${\upsigma}_d^2\textbf{I}$), where ${\upsigma}_d^2$ is the non-additive genetic variance. The model described by Eqs. (2) and (3) is hereafter referred to as model 2. Model 2 was repeated with heterozygosity included as a covariate: this extension is of interest in describing −1× inbreeding depression, but potentially removes non-additive variance and results are given in Supplementary Information 4.

Model 2 provides a direct estimate of the total genetic variance from the ungenotyped trees (${\upsigma}_u^2$) and an indirect estimate from the genotyped trees, which is a function of the additive (${\upsigma}_a^2)$ and non-additive (${\upsigma}_d^2$) genetic variances. In terms of the additive genetic variance, it should be noted that the model parameter ${\upsigma}_a^2$ is *not* the true additive genetic variance of each family since it corresponds to a population with markers in Hardy-Weinberg equilibrium. The true additive genetic variance of each family was therefore estimated by $k{\upsigma}_a^2$, where $k=\overline{\operatorname{diag}\left(\textbf{G}\right)}-\overline{\textbf{G}}$ with the bar denoting the mean value, and *k *= 0.669, 0.686 and 0.731 for SF1, SF2 and SF3 respectively. Note that scaling was not necessary for ${\upsigma}_d^2$ and ${\upsigma}_u^2$ since $\overline{\operatorname{diag}\left(\textbf{I}\right)}-\overline{\textbf{I}}\approx 1$ when the number of genotyped and ungenotyped trees is large. The total genetic variance for the ungenotyped and genotyped trees was then constrained as:

4 $${\upsigma}_u^2=k{\upsigma}_a^2+{\upsigma}_d^2$$

which aligned the ungenotyped and genotyped trees. This constraint was applied when fitting model 2 (see below).

#### Genomic relationship matrix

The genomic relationship matrix, **G**, was constructed separately for each family following Van Raden’s method 1 (VanRaden, 2008), using the alternative allele dosages for each locus for each genotyped tree provided by AlphaPeel (Whalen et al. 2018) following imputation. The dosage is the expected number of alternative alleles accounting for the genotypic probabilities, and takes values between 0 and 2. For example, if the genotype probabilities for locus *j* of tree *i* are 0.01, 0.99 and 0.00 for allele counts 0, 1 and 2, the dosage is 0.99 (0 × 0.01 + 1 × 0.99 + 2 × 0.00). The allele frequencies (*p*_*i*_ for locus *i*) used for centring the dosages and calculating the scalar (∑_*i*_2*p*_*i*_(1 − *p*_*i*_)) were calculated from the full-sib parents for each family for SF1 and SF2 and were either 0.25, 0.50 or 0.75 as each known parent had been genotyped to high coverage and imputed. For SF3, the values of *p*_*i*_ were as observed.

#### Model fitting

Models 1 and 2 were fitted separately for each trait and trial in *ASReml-R*, which obtains REML estimates of the variance parameters and empirical BLUPs of the random effects. Following Tolhurst et al. (2019), the spatial model in Eq. (2) was constructed by fitting a separate model for each master block, with the sets of auto-correlation and variance parameters constrained to be equal across master blocks using the *vcc* argument in *ASReml-R*. Model 2 was fitted with the constraint in Eq. (4) using the ‘own’ function, which constructs user specified variance models. The variance models for the genotyped trees were constructed as var(**u**_2(*a*)_)***=*** ${\sigma}_u^2{r}_a\textbf{G}/k$ and var$\left({\textbf{u}}_{2(d)}\right)={\sigma}_u^2{r}_{\textrm{d}}\textbf{I}$, where ${r}_{\textrm{a}}=k{\sigma}_a^2/{\sigma}_u^2$ and ${r}_{\textrm{d}}={\sigma}_d^2/{\sigma}_u^2$ and ${\sigma}_u^2$ is constrained to equal the total genetic variance of the ungenotyped trees, i.e. var$\left({\textbf{u}}_1\right)={\sigma}_u^2\textbf{I}$.

#### Model summaries

Sample variograms showing the residual semi-variance between plots were constructed after fitting all models to informally assess the spatial models and detect any additional extraneous variation in the column or row directions. An example is presented and summarised in Supplementary Information 3.

Model 2 provided estimates of the fractions of additive and non-additive genetic variance within each family. For SF1 and SF2, these directly estimated the fractions within a full-sib family (*f*_*a*_ and *f*_*d*_), as ${\hat{f}}_a={\hat{r}}_a$ and ${\hat{f}}_d={\hat{r}}_d$. Additional scaling was required for SF3 to account for the two maternal half-sib families, with ${\hat{f}}_a=2.81\ {\hat{r}}_a/\left(3-0.19\ {\hat{r}}_a\right)$ and ${\hat{f}}_d=2.14\ {\hat{r}}_d/\left(2+0.14\ {\hat{r}}_d\right)$. The scaling was calculated using the expected variances across the two families, based on a ratio of 0.594:0.406. Model 2 also provided estimates of broad and narrow-sense heritability, with ${H}^2={\hat{\sigma}}_u^2/\left(\ {\hat{\sigma}}_u^2+{\hat{\sigma}}_v^2+{\hat{\sigma}}_r^2+{\hat{\sigma}}_s^2\right)$ and ${h}^2={\hat{f}}_a{\hat{\sigma}}_u^2/\left(\ {\hat{\sigma}}_u^2+{\hat{\sigma}}_v^2+{\hat{\sigma}}_r^2+{\hat{\sigma}}_s^2\right)$, where the denominator estimated the phenotypic variance, σ_*P*_^2^. The confidence intervals for ${\hat{f}}_a$ were obtained from likelihood profiles calculated by constraining ${\hat{r}}_a$ in model 2 to take values over relevant ranges in the interval [0,1], most densely around the REML estimates. The 95% confidence intervals were defined by the interval for which the drop in 2logL was less than 3.84, the 95% point for χ^2^_1_. Estimates of *f*_*a*_ were pooled across families and sites by summing these profiles. For the estimates of spatial parameters and heritabilities, pooling was done by weighting estimates by the reciprocal of their sampling variance.

## Results

### Imputation

**Fig. 1:**
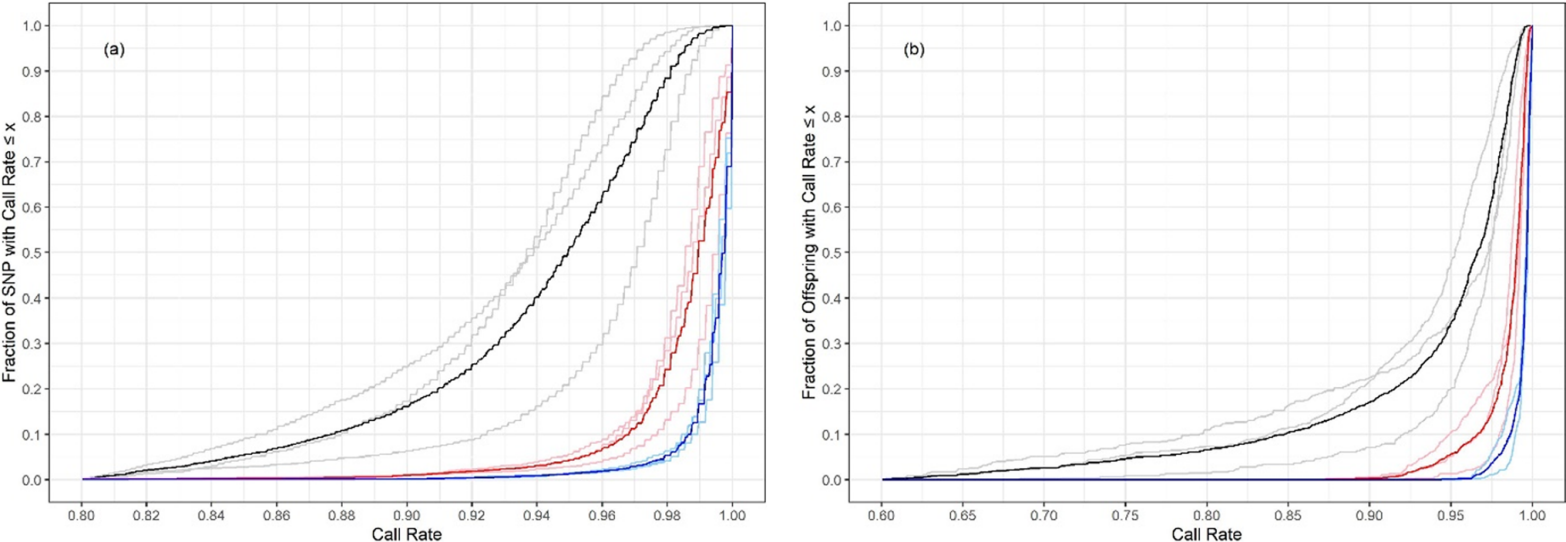
A summary of imputation success rates for sib offspring obtained from AlphaPeel: **a** cumulative distribution function for SNP call rate over offspring and **b** cumulative distribution function for offspring call rate over SNPs. These are shown for no imputation (black), with genotypes assigned with probability >0.7 (blue) and genotypes assigned with probability >0.9 (red), and where light colours are for each family and the dark colour is their average

The cumulative distribution functions of the call rates for SNPs over offspring are shown in Figure 1a, and those for offspring over SNPs in Figure 1b, for no imputation and for different thresholds (*p*) of the posterior probability required to call a genotype following imputation by AlphaPeel. All such functions will tend to 1 as the call rate tends to 1, and if all genotypes were known with certainty, the function would be a step function, where *f(x)*=0 for *x*<1 and *f(x)*=1 for *x*=1. The distribution functions will asymptote towards this step function as the number of genotypes called increases, and the sensitivity of the distribution functions to the value of *p* decreases as confidence in the imputation increases. When the threshold was set to *p=*0.9, 96% of SNPs had call rates exceeding 95% over all offspring (from Figure 1a), and 94% of offspring had call rates exceeding 95% over all SNPs (from Figure 1b). Without imputation, only 50% of SNPs and 67% of offspring had call rates exceeding 95%.

### Spatial parameters

The spatial parameters described in Eq. (2) are treated in this study as nuisance parameters and are summarised below in less detail than the genetic parameters of interest. The sample variograms presented in Supplementary Information 3 show an example outcome from fitting model 2 and illustrate the residual semi-variance between plots *x* rows and *y* columns apart. Variograms peak at the spatial (${\hat{\sigma}}_s^2$) and total error variance (${\hat{\sigma}}_r^2+{\hat{\sigma}}_s^2$), with the discontinuity at zero displacement reflecting the random error variance (${\hat{\sigma}}_r^2$). The general shape of the variograms is determined by the auto-correlation parameters ${\hat{\rho}}_c$ and ${\hat{\rho}}_r$. Table 7 summarises the fraction of random error variance (${\hat{f}}_r$) and the auto-correlation parameters for height at all four ages. Since the ‘column’ and ‘row’ labels were arbitrarily assigned for each site, the estimates ${\hat{\rho}}_c$ and ${\hat{\rho}}_r$ have been pooled into a common estimate $\hat{\rho}$.

**Table 7:** The fraction of random error variance (*f*_*r*_) and the auto-correlation pooled across columns and rows (*ρ*) for height measured at four ages at all three sites (see Eq. (2)). The estimates presented have been averaged across all three families with the range given in parentheses

Age (years)	Huntly		Llandovery		Torridge	
	*f*_*r*_	*ρ*	*f*_*r*_	*ρ*	*f*_*r*_	*ρ*
2	0.85 (0.80, 0.92)	0.85 (0.82, 0.86)	0.88 (0.87, 0.89)	0.77 (0.72, 0.82)	0.73 (0.67, 0.77)	0.90 (0.86, 0.97)
4	0.76 (0.71, 0.85)	0.82 (0.78, 0.83)	0.70 (0.62, 0.76)	0.69 (0.62, 0.80)	0.60 (0.43, 0.76)	0.90 (0.85, 0.95)
6	0.72 (0.63, 0.80)	0.84 (0.83, 0.86)	0.61 (0.57, 0.68)	0.73 (0.67, 0.80)	0.54 (0.40, 0.72)	0.91 (0.86, 0.95)
11	0.52 (0.45, 0.59)	0.91 (0.87, 0.95)	0.53 (0.49, 0.58)	0.81 (0.74, 0.86)	0.60 (0.39, 0.82)	0.93 (0.90, 0.96)

Two trends for height are observed in Table 7: (i) ${\hat{f}}_r$ diminished from 2 to 11 years of age, indicating stronger spatial (positive) associations in height with neighbours as the trees grew; and (ii) the auto-correlations differed between sites, indicating that the observable associations extended over longer distances at Torridge, and conversely smallest at Llandovery. The estimates ${\hat{f}}_r$ and $\hat{\rho}$ for pilodyn depth averaged across families at Torridge were 0.64 (range [0.50, 0.77]) and 0.92 (range [0.86, 0.98]), respectively. For the three measurements of bud burst at 5 years of age (5A, 5B and 5C) for SF1 at the three sites, ${\hat{f}}_r$ was comparatively high (mean 0.81; range [0.73, 0.94]) and $\hat{\rho}$ was also comparatively high (mean 0.78; range [0.61, 0.97]). Taken together, although the common environmental component of variance among neighbours decays slowly for all traits, there is substantial environmental variance independent of neighbours for these ages.

### Pilodyn depth at 10 years

**Fig. 2:**
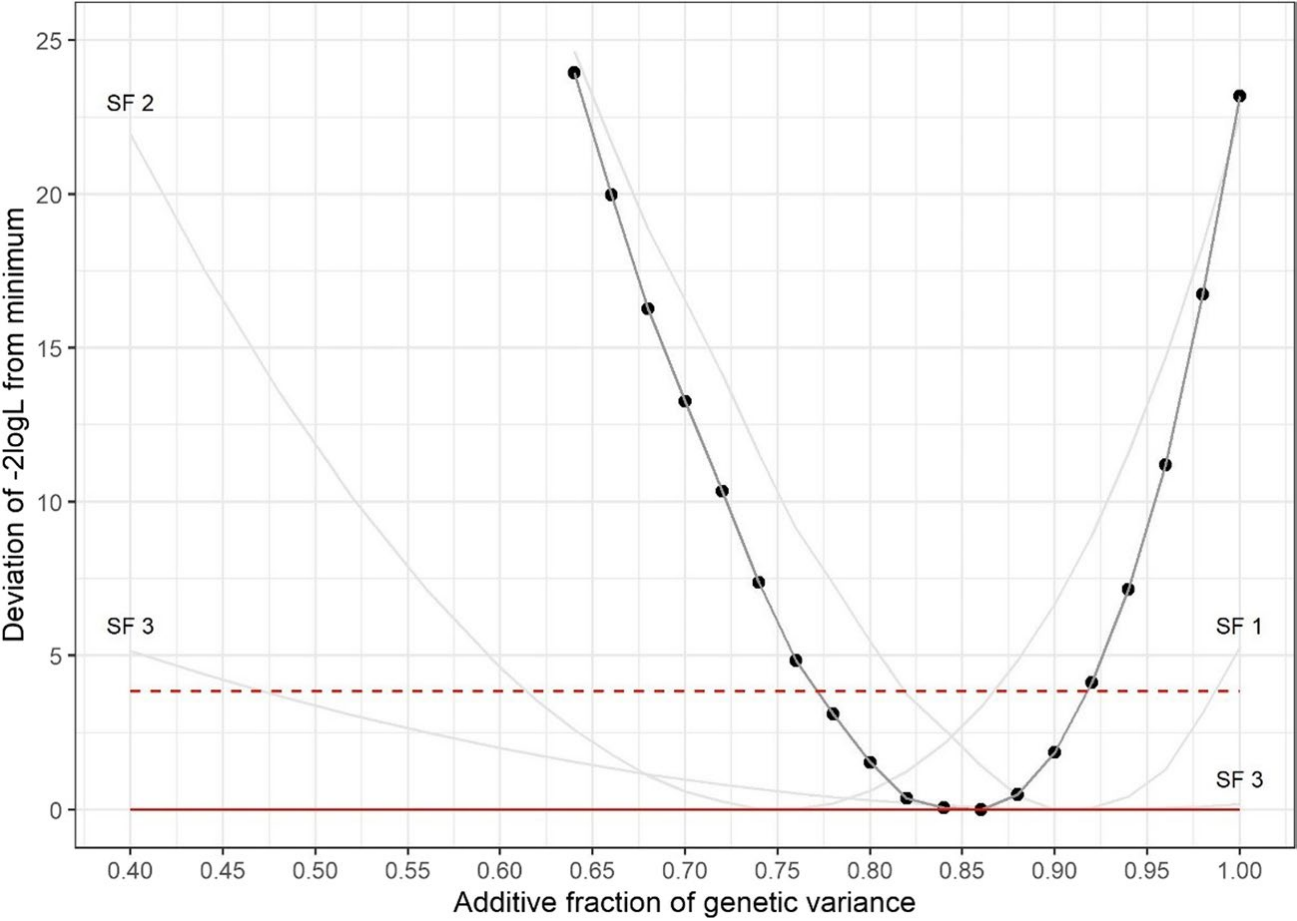
The profiles of −2logL for pilodyn depth measured at 10 years in all three families (grey lines, with labels ‘SF1’, ‘SF2’ and ‘SF3’) according to *f*_*a*_, the fraction of genetic variance that is additive and the profile pooled across families. Each profile has been adjusted by subtracting its minimum value and therefore the junction with the solid red line *y*=0 indicates the maximum likelihood estimate, and the interval below the dashed red line *y*=3.84 indicates the 95% confidence interval

Pilodyn depth was measured at 10 years in all three families at Torridge only, with the results shown in Table 8. The total genetic variance, ${\hat{\sigma}}_u^2$, was considerable in all families, although the broad sense heritability, *H*^2^, differed widely between families (range [0.115, 0.349]). These differences were largely due to the differing environmental variances. Considerable additive genetic variance, ${\hat{\sigma}}_a^2$, was detected in all families with differences in *h*^2^ that reflected the differences in *H*^2^. This correspondence was due to a relative constancy in the fraction of additive genetic variance, ${\hat{f}}_a$. Figure 2 shows the likelihood profile for ${\hat{f}}_a$ in each family, together with the consensus profile pooled across families. The consensus estimate for *f*_*a*_ was 0.84 with 95% confidence interval of [0.77, 0.92]; this estimate was within the 95% confidence intervals for each family, and the hypothesis of a common value across families was not rejected (*P*>0.05; *X*^2^ = 4.75 c.f. *χ*^2^_2_).

**Table 8:** Estimates of the total genetic (*σ*_*u*_^2^) and phenotypic (*σ*_*P*_^2^) variances, broad (*H*^2^) and narrow (*h*^2^) sense heritabilities and the fraction of additive genetic variance (*f*_*a*_) for pilodyn depth measured at 10 years in all three families at Torridge, where *f*_*a*_ for SF3 has been corrected to refer to full-sibs. The associated s.e.s are given in parentheses

Family	*σ*_*P*_^2^	*σ*_*u*_^2^	*H*^2^	*f*_*a*_	*h*^2^
SF1	2.994 (0.125)	1.046 (0.060)	0.349 (0.019)	0.912 (0.043)	0.319 (0.024)
SF2	4.673 (0.368)	1.283 (0.073)	0.275 (0.024)	0.751 (0.064)	0.206 (0.026)
SF3	4.651 (0.650)	0.533 (0.055)	0.115 (0.019)	0.837 (0.128)	0.097 (0.027)

### Bud burst at 5 years

**Fig. 3:**
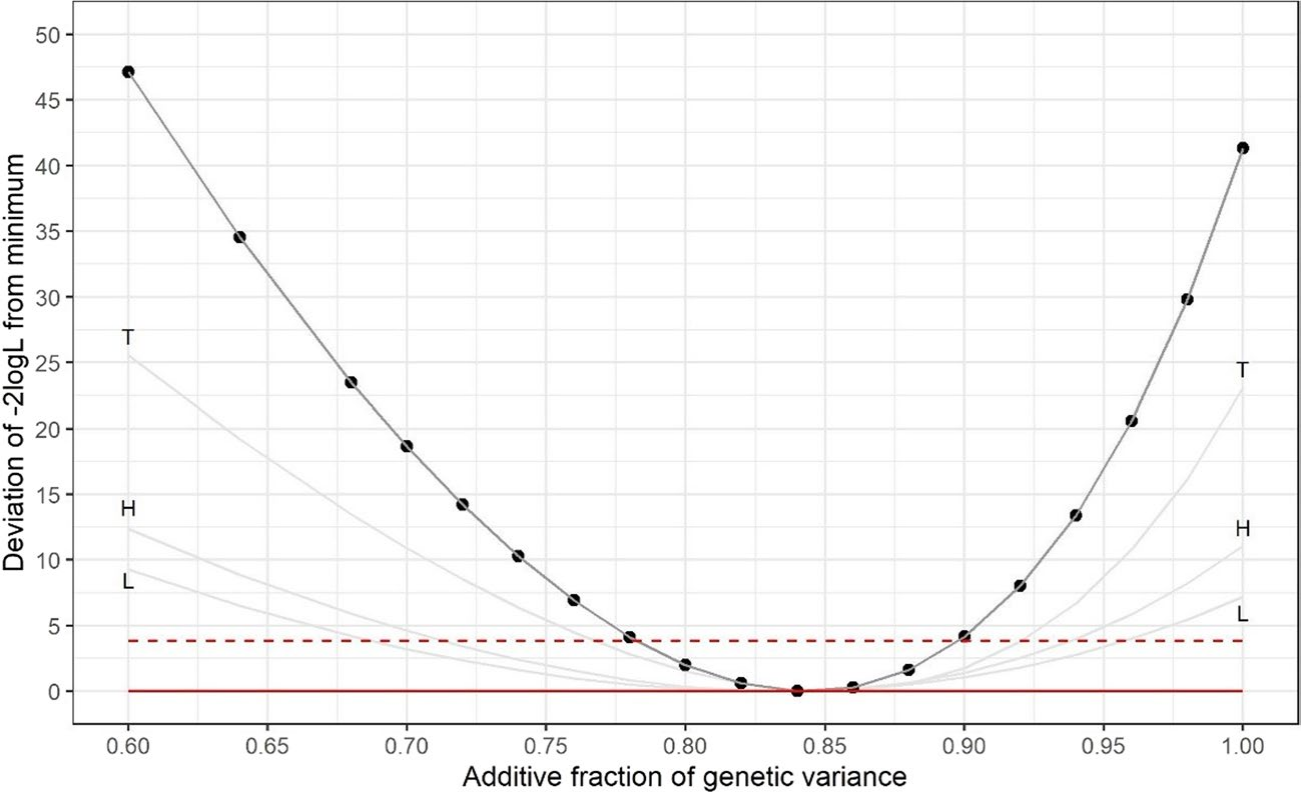
The profiles of −2logL for the first measurement of bud burst at 5 years in SF1 at all three sites (grey lines) according to *f*_*a*_, the fraction of genetic variance that is additive and the profile pooled across sites. Each profile has been adjusted by subtracting its minimum value and therefore the junction with the solid red line *y*=0 indicates the maximum likelihood estimate, and the interval below the dashed red line *y*=3.84 indicates the 95% confidence interval. The labels ‘H’, ‘L’ and ‘T’ denote profiles for Huntly, Llandovery and Torridge respectively

Bud burst at 5 years was only measured in SF1 and Table 9 focuses on the first measurement (5A), and the results for the other two measurements are given in the Supplementary Information 5. Estimates of *H*^2^ differed between sites (range [0.276, 0.476]) and these differences were, again, reflected in the estimates of *h*^2^ for the sites. However, ${\hat{f}}_a$ was very similar across sites. Figure 3 shows the likelihood profiles for ${\hat{f}}_a$ and the consensus profile pooled across sites. The consensus estimate of *f*_*a*_ was 0.83 with 95% confidence interval of [0.78, 0.90]. There was no evidence to reject the hypothesis of a common value across sites (*P*>0.05; *X*^2^ = 0.617 c.f. *χ*^2^_2_). Similar results were obtained for measurements 5B and 5C, which had consensus estimates of 0.91 (s.e. 0.03) and 0.89 (s.e. 0.04) respectively.

**Table 9:** Estimates of the total genetic (*σ*_*u*_^2^) and phenotypic (*σ*_*P*_^2^) variances, broad (*H*^2^) and narrow (*h*^2^) sense heritabilities and the fraction of additive genetic variance (*f*_*a*_) for measurement 5A of bud burst in SF1 at all three sites. The associated s.e.s are given in parentheses

Site	*σ*_*P*_^2^	*σ*_*u*_^2^	*H*^2^	*f*_*a*_	*h*^2^
Huntly	0.251 (0.010)	0.085 (0.005)	0.333 (0.019)	0.836 (0.056)	0.278 (0.026)
Llandovery	0.499 (0.021)	0.138 (0.010)	0.276 (0.024)	0.831 (0.069)	0.229 (0.025)
Torridge	0.699 (0.020)	0.326 (0.018)	0.476 (0.019)	0.851 (0.039)	0.405 (0.025)

### Height at 2, 4, 6 and 11 years

Table 10 shows the broad sense heritabilities, *H*^2^, and phenotypic variances, ${\hat{\sigma}}_P^2$, for height measured at 2, 4, 6 and 11 years in all four families at all three sites. For each site, ${\hat{\sigma}}_P^2$ increased with age but there was no clear trend in the changes in *H*^2^ with age. The estimates of *H*^2^ for Llandovery were generally smaller than for Huntly and Torridge, which were associated with generally larger ${\hat{\sigma}}_P^2$ at a given age compared to the other sites. There were no clear trends in *H*^2^ or ${\hat{\sigma}}_P^2$ between families across ages or sites.

**Table 10:** Estimates of the broad sense heritability (*H*^2^) and phenotypic variance (*σ*_*P*_^2^) for height measured at four ages in all three families at all three sites. The associated s.e.s are given in parentheses

Age (years)	Family	Huntly		Llandovery		Torridge	
		*H*^2^	*σ*_*P*_^2^	*H*^2^	*σ*_*P*_^2^	*H*^2^	*σ*_*P*_^2^
2	SF1	0.202 (0.014)	0.013 (0.000)	0.101 (0.014)	0.015 (0.001)	0.256 (0.015)	0.019 (0.001)
	SF2	0.099 (0.018)	0.014 (0.002)	0.043 (0.011)	0.025 (0.001)	0.286 (0.014)	0.012 (0.001)
	SF3	0.192 (0.014)	0.012 (0.000)	0.103 (0.013)	0.012 (0.000)	0.204 (0.022)	0.009 (0.001)
4	SF1	0.155 (0.013)	0.087 (0.002)	0.088 (0.012)	0.140 (0.006)	0.239 (0.015)	0.130 (0.004)
	SF2	0.165 (0.014)	0.075 (0.004)	0.056 (0.012)	0.178 (0.005)	0.178 (0.015)	0.075 (0.004)
	SF3	0.155 (0.013)	0.097 (0.003)	0.062 (0.012)	0.128 (0.005)	0.064 (0.011)	0.085 (0.008)
6	SF1	0.149 (0.013)	0.341 (0.010)	0.061 (0.012)	0.590 (0.027)	0.225 (0.012)	0.402 (0.014)
	SF2	0.174 (0.013)	0.214 (0.005)	0.065 (0.012)	0.544 (0.016)	0.222 (0.015)	0.324 (0.017)
	SF3	0.154 (0.012)	0.288 (0.009)	0.097 (0.015)	0.358 (0.033)	0.081 (0.011)	0.368 (0.017)
11	SF1	0.089 (0.012)	1.416 (0.134)	0.056 (0.011)	1.786 (0.055)	0.214 (0.015)	0.779 (0.029)
	SF2	0.100 (0.028)	1.895 (0.505)	0.062 (0.011)	2.156 (0.136)	0.334 (0.027)	1.282 (0.097)
	SF3	0.196 (0.014)	1.607 (0.067)	0.076 (0.018)	1.341 (0.251)	0.180 (0.015)	1.465 (0.079)

The consensus estimates of *f*_*a*_ pooled across families are given in Table 11 for each age and site. There was broad evidence of increasing ${\hat{f}}_a$ with age at all sites, in particularly ${\hat{f}}_a$ was largest at 11 years and smallest at 2 years. This trend was particularly evident in the consensus estimates of *f*_*a*_ after pooling across sites with ${\hat{f}}_a$ increasing from 0.60 at 2 and 4 years to 0.75 at 11 years, with 95% confidence intervals with only small overlap. The confidence interval for the consensus estimate of *f*_*a*_ at 11 years does not include 1, i.e. not all genetic variance is additive. However, some individual families at some individual sites do include 1 in their confidence intervals, which are wider, and the best point estimate for SF2 at 11 years at Llandovery was 1. There was no overall evidence of differences between families across sites from the goodness of fit tests (*P*>0.05, *X*^2^=34.325, c.f. *χ*^2^_24_), although Torridge at 4 and Huntly at 11 years of age gave isolated significance (*P*<0.05, *X*^2^=4.81 and 4.42 respectively, c.f. *χ*^2^_2_).

**Table 11:** The fraction of additive genetic variance (*f*_*a*_) for height measured at four ages at all three sites. The estimates presented are pooled across families using likelihood profiles, and the consensus estimate is obtained by pooling the resulting profiles across sites. The associated 95% confidence intervals are given in parentheses

Age (years)	Huntly	Llandovery	Torridge	Consensus
2	0.54 (0.40, 0.67)	0.52 (0.32, 0.73)	0.62 (0.51, 0.73)	0.58 (0.48, 0.68)
4	0.59 (0.46, 0.73)	0.59 (0.36, 0.85)	0.67 (0.55, 0.79)	0.63 (0.52, 0.74)
6	0.60 (0.46, 0.73)	0.63 (0.43, 0.93)	0.72 (0.62, 0.82)	0.67 (0.57, 0.77)
11	0.62 (0.49, 0.74)	1.00 (0.69, 1.00)	0.79 (0.70, 0.86)	0.75 (0.66, 0.83)

## Discussion

This study combines all available phenotypic and genomic data from a multi-site, clonally replicated experiment with large sib families produced by controlled crossing to partition the genetic variance observed between clones for height, bud burst and pilodyn penetration depth into additive and non-additive components. The additive genetic variance formed the largest fraction of total genetic variation for all traits, with estimates of 0.58 for height at 2 years of age increasing to 0.75 at 11 years, 0.84 for pilodyn penetration depth at 10 years and ranging from 0.83 to 0.91 for the 3 measures of bud burst at 5 years. This partition is possible as the model underlying the Van Raden relationship matrix, **G**, is a ridge regression model on marker allele counts and therefore only describes what is observed as an additive sum of effects over loci, whereas the total genetic variance obtained from the clonal replication includes dominance and epistasis. The experimental design had several aspects that made the study feasible, or more powerful, beyond the clonal replication of the offspring. Firstly, the experiment’s large sib families made it possible to consolidate genotypes obtained from RADseq by imputation, using the recent availability of a molecular map for Sitka spruce (Tumas et al. 2023). Secondly, the measurement of traits across sites, or across families, or both, allowed for replicated estimates of *f*_*a*_, and the estimates were found to be very largely consistent, subject to their sampling errors. The size of the sib families also gave the opportunity to manage the unexpected parentage of SF3.

An important theoretical perspective to consider when comparing the current results with those from other published studies is that here the parameter reported (*f*_*a*_) is defined within full-sib families, even though this required an adjustment in SF3. Therefore, the estimates presented here are partitions of the Mendelian sampling, and not the full genetic variance for a random-mating population, which may be obtained from other approaches. The expectations for the additive and non-additive components can be scaled up to the corresponding variance for a full random-mating population and, based on these expectations, the fraction *f*_*a*_ would increase. Although half the additive variation lies within families, a greater portion (3/4) of the dominance and the additive × additive epistatic variation is within families, and more than 3/4 for higher order epistatic terms (Falconer & Mackay, 1996). Assuming that any non-additive variation observed within families is explained by dominance or additive by additive, then the expectation is that *f*_*a*_ in this study corresponds to 3 *f*_*a*_ /(2+ *f*_*a*_) in a random mating population, e.g. *f*_*a*_ = 0.6 and 0.8 corresponds to 0.69 and 0.86. While only three sib families were sampled, the consensus estimates for *f*_*a*_ for the traits measured on all families is important in providing guidance on the wider population.

Among previous studies, Weng et al. (2008) estimated partitions of genetic variance in white spruce, a close relative to Sitka spruce, for a similar range of ages for height, and also for pilodyn depth. Their results show comparable estimates of *f*_*a*_ ranging from ~ 0.4 at 4 years to 0.8 at 14 years, despite the large s.e.s found in their data. The study of Nguyen et al. (2022) in Norway spruce covered a range of ages for height between 6 and 12 years and their results also appear to suggest that *f*_*a*_ decreases between these ages; however, examination of the results shows large s.e.’s and negative estimates of *f*_*a*_ which seriously limit interpretation. Results for Norway spruce were also reported by Chen et al. (2019) using genomic analysis: for height at 17 years, *f*_*a*_ ~ 0.4 and 0.6 at two sites, and for pilodyn depth at 30 years of age *f*_*a*_ ~ 1 at both sites. Among other studies of height, Isik et al. (2003) assessed four ages between 1 and 6 years and Baltunis et al. (2007) at 2 years, both in loblolly pine, Baltunis et al. (2013) at 12 years in yellow cypress but for the large sampling errors limit comparability. Few studies have examined pilodyn depth, but those that have are in agreement with the findings here that the fraction of additive genetic variance is very high, with estimates of 0.90 (s.e. 0.18) at 26 years of age in white spruce (Nguyen et al. 2022); ~0.8 in *Eucalyptus globulus* at 4 years derived from the results of Costa de Silva et al. (2004). There are no comparable results for bud burst in other published studies. Each trait should be expected to have its own architecture, but too few results are available to attempt generalisation particularly given the substantial standard errors of many estimates (stem diameter in Norway spruce (Nguyen et al. 2022; Berlin et al. 2019), *Eucalyptus globulus* (Costa de Silva et al. 2004) and radiata pine (Baltunis et al. 2009); wood quality traits in white spruce (Nguyen et al. 2022) and Norway spruce (Chen et al. 2019)).

This study partitions the genetic variance in Sitka spruce into additive and non-additive components using an approach similar to that of de Almeida Filho et al. (2019), which used the classical ridge regression model to estimate the fraction of additive genetic variance and the clonal variance to estimate the total genetic variance. However, their approach requires all trees to be genotyped and removes any ungenotyped trees. In this paper, a linear mixed model was developed which combines all available phenotypic and genomic data on all trees, regardless of whether they have been genotyped. In particular, the fraction of additive genetic variance was estimated using the subset of genotyped trees and the total genetic variance estimated using all genotyped and ungenotyped trees. This approach preserves all available data to estimate the genetic and non-genetic variances, which is particularly important for spatial modelling (as it requires a continuous spatial structure). It is equivalent to setting the ungenotyped trees as diagonal (independent) in the genomic relationship matrix within model 2, so that the additive component for these trees would not be well defined. There are also similarities to single-step GBLUP (Legarra et al. 2009), but without the need for pedigree or the need to construct an **H** matrix. The distinguishing feature here is that the primary goal of this study was to obtain reliable estimates of the fraction of additive genetic variance, rather than obtaining predictions of additive genetic merit for genomic selection. Furthermore, single-step GBLUP involves setting an equivalence between the genetic variances being estimated, whereas here the data are used to estimate to what extent the variances coincide.

Obtaining reasonable precision on the fraction of additive genetic variance using pedigree alone has proved challenging as it typically involves scaling up and calculating linear functions of the estimated pedigree components. The models used here are parsimonious in that no attempt has been made to partition the non-additive genetic variance into dominance and epistatic components to avoid overfitting. The further partition is in general feasible, without assuming Hardy-Weinberg equilibrium, as shown by Vitezica et al. (2017), and exemplified in Nile tilapia (*Oreochromis niloticus*) by Joshi et al. (2020). This involves using the markers to calculate orthogonal relationship matrices for the dominance and epistatic components (e.g. the additive by additive relationship matrix is proportional to the Hadamard product of G with itself). The partition was attempted in the study of Chen et al. (2019) in Norway spruce but assumed Hardy-Weinberg equilibrium. Furthermore, no attempt has been made here to estimate genetic variance across families (as distinct from pooling the results within families) for two reasons: (i) the number of parents is small and (ii) the number of markers are too few for satisfactory estimation across families, but more than adequate within families (Lillehammer et al. 2013). This leads to limitations in interpretation of this study, e.g. there is no assessment of whether additive marker effects in one family are similar to those from another, despite the consistency of the *f*_*a*_ observed within families. .

The evidence suggests that the fraction of additive genetic variance increases with age for height towards the high fractions observed for pilodyn and bud burst. The estimates for results of Supplementary Information 4 also show no evidence of inbreeding depression for any of the traits and therefore no evidence on the form of the non-additive genetic variance, e.g. in the study of Joshi et al. (2020) in Nile tilapia, the extra genetic variance observed in full-sibs aligned with additive by additive epistasis and not dominance. While the form of the non-additive genetic variance may be less relevant for deployment strategies using clones, it does influence the form of the breeding programme, as additive by additive fractions become converted to additive variance under selection and little benefit is expected from establishing sub-lines for crossing, as in reciprocal recurrent selection.

Knowing the relative proportions of additive and non-additive genetic variance is important in deciding how to proceed with the Sitka spruce breeding programme. If genetic variation is predominantly additive, it can make the breeding process somewhat more straightforward and predictable; the greatest genetic gain is made combining those parents with the largest estimates of breeding value for the traits subject to selection. A low proportion of non-additive genetic variation reduces the likelihood of exceptional family combinations resulting from a mating of two parents of moderate or poor breeding values. The breeder can now simplify the breeding process through the use of elite breeding programmes such as ‘Nucleus Breeding’ as originally suggested for sheep breeding (Jackson and Turner, 1972) and adapted for trees by Cameron et al. (1989). The indications from this study suggest that elite breeding could be used to good effect in the Sitka spruce breeding programme to maximise early increases in height, selection for timing of bud burst avoiding the more frost-damage prone, early flushing genotypes and pilodyn-pin penetration.

### Supplementary information

ESM 1: (DOCX 1276 kb)

## Data Availability

All data will be made available on reasonable request. Some data is already in data repositories and remaining data will be lodged on publically accessible data repositories.
