# Supplementary material for: Additive and non-additive genetic variance in juvenile Sitka spruce (Picea sitchensis Bong. Carr)
Source: Tree Genet Genomes. 2023 Nov 8;19(6):53. doi: 10.1007/s11295-023-01627-5 (PMC10632294; doi:10.1007/s11295-023-01627-5)
Supplement: Supplementary file 1 — (DOCX 1276 kb) [file 11295_2023_1627_MOESM1_ESM.docx]

**Supplementary Information 1.**  The sib structure of SF3.

**Detection of two full-sib families.**

RADseq genotyping is based upon a random number of sequence reads at set of loci defined by the restriction enzymes used in the assay. A consequence of the stochasticity is a background number of loci at which the genotype of the offspring and parent will be called homozygotes for different alleles; this number will decrease as the coverage of both offspring and parent increases. The number of opposing homozygotes was examined for parent-offspring pairs, separately for each pair, in SF3 using 5975 SNPs identified as both segregating in SF3 and subsequently placed on the linkage map of Tumas et al. (2023), i.e. 45% of those recorded for SF3 in Table 5. No imputation was undertaken beforehand. Fig S1.1 shows the distributions of the counts and displays a marked bimodal distribution for the nominated paternal parent of SF3, but not for its maternal parent. This raised the possibility that the offspring were a mixture of families with a common maternal parent. SF3 was split into 2 sub-groups A and B according to the number of opposing homozygotes: A <200, B>200. Sub-group A was consistent with full-sibs from the nominated parents in the design. Of the 478 genotyped offspring 284 were in A and 194 in B, which implies among all 1496 offspring, from which those genotyped were randomly sampled, a fraction 0.594 (s.e. 0.022) were full-sibs from the nominated parents.

It was hypothesised that sub-group B constituted a distinct family of full-sibs bred from an unknown paternal parent, as none of the other genotyped parents (of SF1 and SF2) were compatible as the paternal parent of B. To test this hypothesis the genomic relationship matrix **G** was calculated for all individuals in SF3 following Method 1 of Van Raden (2008) and the distribution of the elements was plotted according to sub-groups. Fig. S1.2 shows the distributions of the relationships are consistent with the hypothesis of 2 full-sib families.

**Testing the 2 full-sib family hypothesis.**

To test the hypothesis the genotypes obtained for the 1630 ‘common’ and mapped SNPs were used for all those genotyped in SF3, and the parents of SF1 and SF2. The genotypes were prior to imputation. Firstly, as above, the genomic relationship matrix **G** was calculated for all individuals in SF3, and the distribution of the elements was plotted according to sub-groups A and B; see Fig S1.3(a).

The genomes of the offspring were then simulated according to 2 scenarios from the known genotypes of all the 6 known parents. Only a randomly chosen subset of 200 loci were used as the loci were treated as unlinked in the simulation and so would otherwise lengthen the genome compared to the 1630 loci in 12 linkage groups, and reduce the variation in relationships (Vissher et al. 2006). Among the 6 genotyped parents any missing genotypes replaced by randomly sampled genotypes. In the first scenario sub-group A was simulated by the nominated parents for SF3, and sub-group B was simulated from the nominated maternal parent and a single paternal parent randomly chosen from among the 4 parents of SF1 and SF2 for all offspring. In the second scenario, sub-group B was simulated with the paternal parent of each offspring randomly sampled from the 4 parents of SF1 and SF2 . Typical outcomes are shown in Fig S1.3(b) and Fig S1.3(c).

The conclusion from the simulation was that the evidence strongly supported the hypothesis that sub-group B was a single full-sib family, as any mix of parentage showed a much greater spread of relationships within sub-group B.

**References**

VanRaden PM (2008) Efficient methods to compute genomic predictions. J Dairy Sci, 91:4414-4423. <http://doi>.org/10.3168/jds.2007-0980.

Visscher PM, Medland SE, Ferreira MAR, Morley KI, Zhu G et al. (2006) Assumption-free estimation of heritability from genome-wide identity-by-descent sharing between full-siblings. PLoS Genetics, 2:e41. http://doi.org/10.1371/journal.pgen.0020041

**Fig S1.1** The distributions of the number of opposing homozygotes between nominated parents and offspring in SF3, where red denotes frequency for maternal parent, and blue for paternal parent.

**
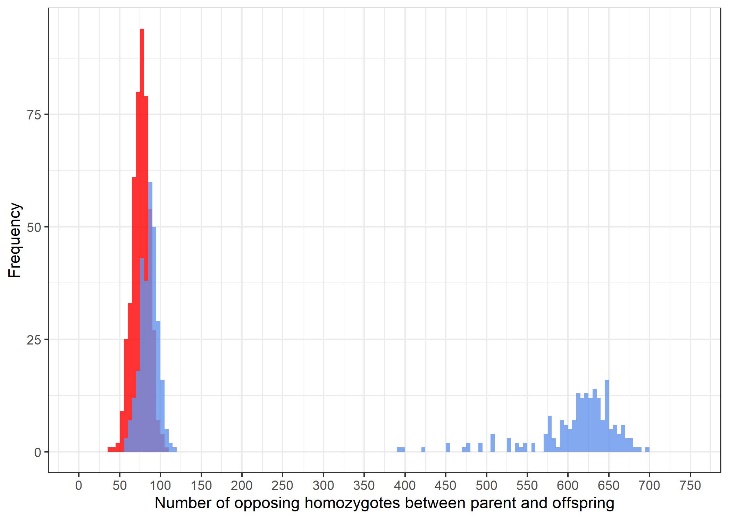
**

**Fig S1.2** The distribution of the genomic relationship coefficients, calculated following method 1 of Van Raden (2008) for the putative two full-sib families A and B. Off-diagonal elements: (A,A) red; (B,B) pink; (A,B) blue. Diagonal elements: A, green; B, gold.

**
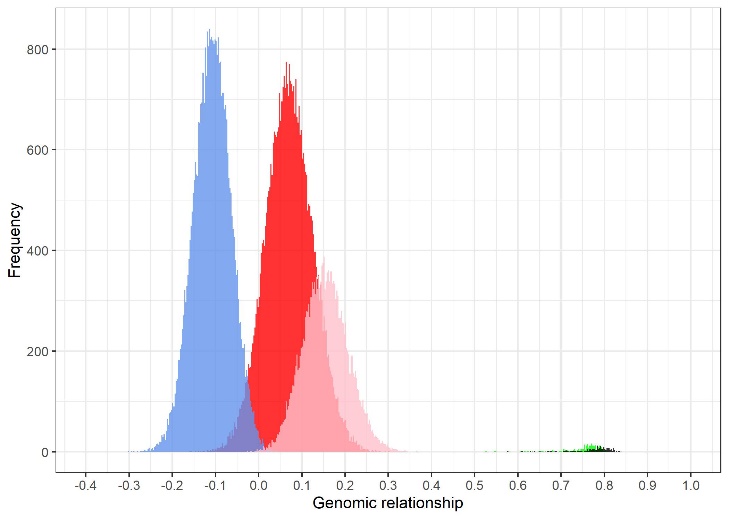
**

**Fig S1.3** The distribution of genomic relationship coefficients for SF3 using (a) the 1630 ‘common’, mapped SNPs, (b) using a subset of 200 SNPs and simulated as unlinked with B as full-sibs, (c) as (b) but with B treated as a mix of families with different paternal parents. Off-diagonal elements: (A,A) red; (B,B) pink; (A,B) blue. Diagonal elements: A, green; B, gold.

**
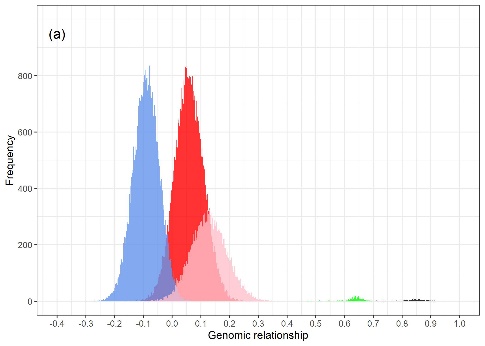

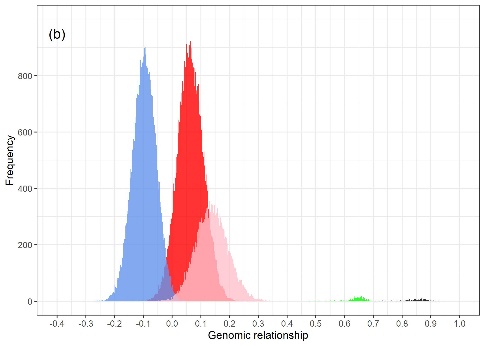

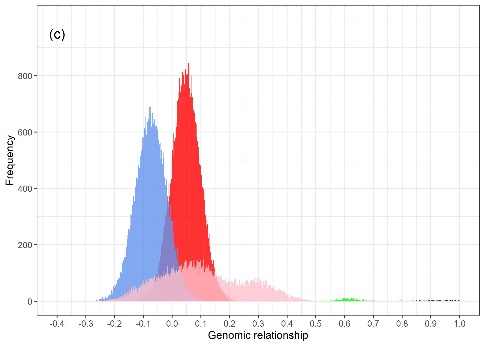
**

**Supplementary Information 2.**  Assessment of accuracy of genotype assignment in SF3.

The accuracy of imputation was additionally assessed using a trial involving the masking of genotypes in two stages using the 1630 ‘common’ and mapped SNPs. For these SNPs over the 478 genotyped offspring the fraction of missing genotypes was 0.040.

In the first stage 3 SNPs from each offspring and each linkage group, all with assigned genotypes were randomly sampled (i.e. a total of 17208 SNPs). These assignments prior to imputation were then compared to the posterior probability for the genotype after imputation. The mean posterior probability was 0.990. In the second stage, the sampling protocol was repeated and these assignments were masked prior to imputation (i.e. as if missing genotypes). The outcomes were compared as before and the mean posterior probability of the masked genotypes was lower 0.921.

These results give an indication of genotype uncertainty errors by weighting the trial outcomes with the fraction of SNPs missing: i.e. 0.960 $\times$ (1-0.990) + 0.040 $\times$ (1-0.921) = 0.013.

**Supplementary Information 3.**  Examples of trial layouts and sample variograms.

Fig S3.1 shows an example of how master blocks were constructed for spatial modelling. There are two important features of the trial layouts shown. Firstly, the layout in (a) contains four replicate blocks arranged in a single contiguous array. This trial is treated as having a single “master block”, defined by all replicate blocks in the trial. Secondly, the layout in (b) contains two groups of replicate blocks that are spatially separated (non-contiguous). Two master blocks were constructed with each master block defined by two replicate blocks. The important feature here is that there is an unknown distance between master blocks, which prevents the construction of a single contiguous array. Instead, separate spatial models were fitted to each master block following Tolhurst et al. (2019). The auto-correlation and variance parameters were then constrained to be equal across master blocks during model fitting. This provides a flexible and parsimonious framework to handle non-contiguous arrays in spatial modelling

Fig S3.2 shows the sample variograms obtained for height measured at 11 years in family SF1 at Huntly from fitting Model 2, which includes the residual spatial model in Eqn 2. This example was chosen as the fraction of random error variance was low (f_r_ = 0.45) and the auto-correlation pooled across columns and rows was high (ρ=0.95). The sample variograms are constructed using the residual semi-variance between plots *x* rows and *y* columns apart, and are hence different to a theoretical variogram constructed directly from the column and row auto-correlation parameters, ρ_c_ and ρ_r_ (Gilmour et al. 1997). There are three important features of the sample variograms shown. Firstly, the variograms have properties determined by the parameters: Fig. 3.1 (a) peaks at ~0.7 which reflects the spatial error variance (σ_s_^2^); and Fig. 3.1 (b) peaks at ~1.3 which reflects the total error variance (σ_r_^2^ + σ_s_^2^). The discontinuity at zero displacement in (b) is ~0.6 which reflects the random error variance (σ_r_^2^). Secondly, both variograms demonstrate a substantial increase in semi-variance between plots either 60 columns or 60 rows apart. Random column and row terms were fitted to account for this variation, but these proved unsuccessful. Lastly, there is a noticeable decrease in semi-variance between plots 11, 22, 33, … rows apart. Unfortunately, the source of this variation could not be identified. Standard random row terms were supplemented with terms cycling 1 to 11, instead of one to the number of rows, but this proved unsuccessful.

**References**

Gilmour AR, Cullis BR, Verbyla AP (1997) Accounting for natural and extraneous eariation in the analysis of field experiments. J Agric Biol Environ Stat 2:269-293. <https://doi.org/10.2307/1400446>

Tolhurst, DJ, Mathews, K, Smith, AB, Cullis, BR (2019) Genomic selection in multi‐environment plant breeding trials using a factor analytic linear mixed model. J Anim Breed Genet 136:279–300. https:// doi.org/10.1111/jbg.12404

**Figure S3.1** Examples of trial layouts. Plot (a) shows a trial with a single master block and (b) shows a trial with two master blocks. Note that there is an unknown distance between master blocks in (b), which requires constraints to be applied during spatial modelling (described in text). Replicate blocks are distinguished with thin black lines.

Row

Column

(a)

(b)

Unknown distance

Master block 1

Master block 2

Replicate A

Replicate B

Replicate C

Replicate D

Replicate C

Replicate D

Replicate A

Replicate B

**Figure S3.2** Sample variograms for height measured at 11 years in SF1 at Huntly. Plot (a) shows the variogram for the spatial trend and (b) shows the variogram for the spatial trend plus random error. The z-axis shows the residual semi-variance between pairs of plots *x* rows and *y* columns apart, with shading changing from red to green with increasing magnitude. Only semi-variances based on more than 50 pairs are shown.

**Supplementary Information 4.** Regression on observed heterozygosity.

**Methods.** Model 2 was modified to include the heterozygosity of the offspring as a linear covariate, and it was fitted separately to all site by family by trait combinations. For tree heights and pilodyn penetration depth the coefficients were pooled across families within sites following Dersimian and Laird (1986). The pooled estimate for height at each age, and the estimates for bud burst observations in Family 1 were then pooled over sites using DerSimian and Laird (1986).

**Results.**  The regression coefficients are shown in Table S4.1. Since the covariate was heterozygosity positive values represent deleterious inbreeding depression. There was no evidence of heterogeneity when pooling across families for height or pilodyn. Similarly, there was no evidence of heterogeneity when pooling across sites. The magnitudes of the consensus estimates rarely exceeded 1 s.e. and were always <1.3 s.e. In summary, there was no evidence for an effect of heterozygosity and inbreeding depression in these data.

**Table S4.1.** The regression coefficient for the fraction of heterozygous marker loci for height, bud burst and pilodyn. Coefficients were pooled across families, and consensus estimates across sites were calculated following DerSimian and Laird (1986). The s.e.s are in parentheses.

|  |  | Site | | | | | |  | |
| --- | --- | --- | --- | --- | --- | --- | --- | --- | --- |
| Trait | Age | Huntly | | Llandovery | | Torridge | | Concensus | |
| Height | 2 | 0.01 | (0.10) | 0.03 | (0.11) | -0.01 | (0.09) | 0.01 | (0.06) |
|  | 4 | -0.37 | (0.31) | -0.25 | (0.33) | 0.11 | (0.26) | -0.13 | (0.17) |
|  | 6 | -1.01 | (0.74) | 0.35 | (0.62) | -0.27 | (0.77) | -0.23 | (0.40) |
|  | 11 | -1.76 | (1.06) | -0.16 | (1.21) | -0.29 | (1.00) | -0.76 | (0.62) |
| Bud Burst * | 5A | 1.52 | (0.91) | -1.70 | (1.38) | 2.56 | (1.62) | 0.79 | (1.18) |
|  | 5B | 0.39 | (1.12) | -0.18 | (1.32) | 2.75 | (2.14) | 0.51 | (0.79) |
|  | 5C | 1.19 | (0.95) | -0.62 | (1.10) | 1.51 | (1.96) | 0.55 | (0.68) |
| Pilodyn | 10 |  |  |  |  | 2.81 | (2.44) |  |  |

**Reference**

DerSimonian R, Laird N (1986) Meta-analysis in clinical trials. Control Clin Tr 7:177-188. <http://doi.org/10.1016/0197-2456(86)90046-2>

**Supplementary Information 5**. Results for bud burst observations 5B and 5C.

The 5B and 5C measurements of bud burst in SF1 were subject to the same analyses as 5A, and the results for 5A are presented in the main text. The Tables S5.1 and S5.2 show the results for 5B and 5C corresponding to Table 9 for 5A. The consensus estimates across sites, obtained using Dersiminian & Laird (1986), were 0.909 (s.e. 0.027) and 0.891 (s.e. 0.037) for 5B and 5C respectively.

**Table S5.1** Estimates of the total genetic (σ_u_^2^) and phenotypic (σ_P_^2^) variances, broad (H^2^) and narrow (h^2^) sense heritabilities and the fraction of additive genetic variance (f_a_) for measurement 5B of bud burst in SF1 at all three sites. The associated s.e.s are given in parentheses.

| Site | σ_P_^2^ | σ_u_^2^ | H^2^ | f_A_ | h^2^ |
| --- | --- | --- | --- | --- | --- |
| Huntly | 0.475 (0.029) | 0.077 (0.006) | 0.163 (0.015) | 0.867 (0.092) | 0.141 (0.020) |
| Llandovery | 0.499 (0.019) | 0.142 (0.010) | 0.285 (0.018) | 0.939 (0.063) | 0.268 (0.024) |
| Torridge | 1.288 (0.044) | 0.628 (0.034) | 0.488 (0.018) | 0.907 (0.032) | 0.442 (0.024) |

**Table S5.2** Estimates of the total genetic (σ_u_^2^) and phenotypic (σ_P_^2^) variances, broad (H^2^) and narrow (h^2^) sense heritabilities and the fraction of additive genetic variance (f_a_) for measurement 5C of bud burst in SF1 at all three sites. The associated s.e.s are given in parentheses.

| Site | σ_P_^2^ | σ_u_^2^ | H^2^ | f_A_ | h^2^ |
| --- | --- | --- | --- | --- | --- |
| Huntly | 0.385 (0.025) | 0.050 (0.005) | 0.129 (0.014) | 0.901 (0.116) | 0.117 (0.019) |
| Llandovery | 0.381 (0.009) | 0.061 (0.006) | 0.159 (0.015) | 0.846 (0.116) | 0.135 (0.021) |
| Torridge | 1.267 (0.190) | 0.408 (0.023) | 0.322 (0.050) | 0.895 (0.041) | 0.289 (0.047) |

**Reference**

DerSimonian R, Laird N (1986) Meta-analysis in clinical trials. Control Clin Tr 7:177-188. http://doi.org/10.1016/0197-2456(86)90046-2
